# A Fluorescent Cy7-Mercaptopyridine for the Selective Detection of Glutathione over Homocysteine and Cysteine

**DOI:** 10.3390/s18092897

**Published:** 2018-09-01

**Authors:** Shin A Yoon, Wantae Kim, Amit Sharma, Peter Verwilst, Miae Won, Min Hee Lee

**Affiliations:** 1 Department of Chemistry, Sookmyung Women’s University, Seoul 04310, Korea; dbstlsdk23@sookmyung.ac.kr; 2 School of Chemical and Biological Engineering, Seoul National University, Seoul 151-742, Korea; wtkim223@naver.com; 3 Department of Chemistry, Korea University, Seoul 02841, Korea; amitorg83@gmail.com (A.S.); peterverwilst@hotmail.com (P.V.); miaewon@korea.ac.kr (M.W.)

**Keywords:** NIR fluorescent probe, Cy7 dye, glutathione (GSH), 2-mercaptopyridine

## Abstract

We describe a near-infrared (NIR) fluorescent probe **1** for the selective detection of GSH over Hcy and Cys under physiological conditions. Probe **1** was composed of Cy7 as a NIR dye and 2-mercaptopyridine as a GSH-reactive site and fluorescence quencher. In the presence of GSH, the 2-mercaptopyridine functionality of probe **1** was replaced by the thiolate group of GSH through a nucleophilic substitution reaction with a fluorescence increase at 818 nm. The probe was found to be highly selective for GSH over Hcy, Cys, and other tested potential interferants, including ROS and metal ions. In addition, probe **1** successfully displayed fluorescence changes in response to changing the GSH concentrations in MDA-MB-231 cells in the presence of external agents i.e., *N*-acetyl-l-cysteine (NAC; as GSH inducer) or buthionine sulfoximine (BSO; as GSH inhibitor). We envision that probe **1** will serve as a promising sensing tool for monitoring the changes of the GSH level and the understanding of the roles of GSH under physiological and pathological conditions.

## 1. Introduction

Bio-thiols, e.g., glutathione (GSH), cysteine (Cys), and homocysteine (Hcy), play important roles in maintaining the redox status of biological systems [1,2]. In humans, GSH is the most abundant non-protein cellular thiol, with concentrations ranging from 0.5 to 15 mM [3]. GSH with its oxidized partner (GSSG) is involved in several crucial biological processes, including xenobiotic metabolism, gene regulation, signal transduction, growth, stress responses, immune responses [4,5,6]. These functions are dynamically controlled by the intracellular distribution and concentration of GSH. In addition, abnormal levels of GSH are associated with cancer, heart disorders, aging and other diseases. Consequently, the detection and imaging of intracellular GSH is of great demand for the deep understanding of GSH-related pathophysiological investigations, diagnosis and identification of related diseases.

To date, a number of artificial fluorescent probes that can detect bio-thiols with high temporal and spatial resolution in living systems have been exploited [7,8,9,10]. However, most of the probes cannot discriminate GSH from Hcy/Cys. Because their sensing mechanisms, such as Michael addition, cleavage of sulfonamide and sulfonate esters, and disulfide exchange, etc., are based on nucleophilicity of thiols. In addition, most probes were constructed by fluorophores, e.g., coumarin, naphthalimide, rhodamine, that absorb and emit the lights in the UV-Vis region, which can damage living cells and be affected by the autofluorescence of biomolecules. Recently, Yang and his co-workers exploited a monochlorinated BODIPY-based probe for discrimination of GSH over Hcy/Cys [11]. The chlorine of BODIPY was replaced by thiolates of GSH and Hcy/Cys, and subsequently the amino groups of Hcy/Cys, but not GSH, replaced the sulfur to form amino-substituted BODIPY, hence resulting in different photophysical changes towards GSH and Hcy/Cys. The chemical ligation was extensively employed as a powerful method for the discrimination of GSH over Hcy/Cys [12,13,14,15,16,17]. In addition, the near-infrared (NIR) fluorescent dyes are good candidates for detecting GSH as they can provide several merits, such as no interference from the auto-fluorescence of biomolecules, minimization of cellular photodamage, etc., in the field of bioimaging research [18,19,20,21,22,23,24,25]. Particularly, cyanine-based NIR dyes are suitable for bioimaging due to their proper excitation and emission wavelengths as well as their biocompatibility [26]. However, the NIR fluorescent probes for the selective detection of GSH over Hcy and Cys are still rarely reported [18,19,20].

**Scheme 1:**
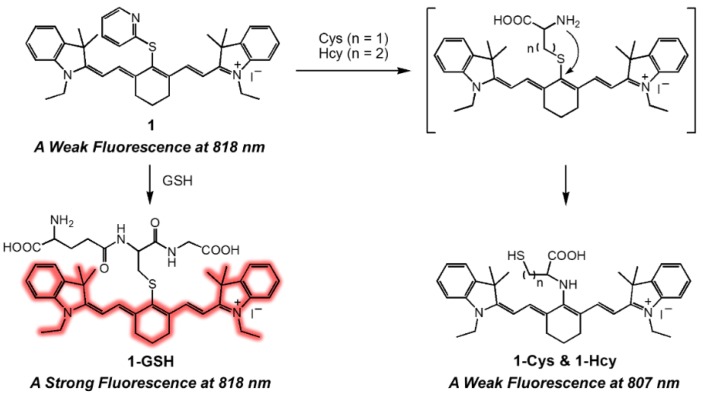
Proposed detection mechanism of **1** to GSH over Hcy/Cys.

In the current work, we present a NIR fluorescent probe **1** that can selectively detect GSH over Hcy and Cys under physiological conditions. Probe **1** was comprised of Cy7 as a NIR fluorescent dye and 2-mercaptopyridine as a GSH-reactive site and fluorescence quencher. As proposed in Scheme 1, probe **1** displayed a weak fluorescence at 818 nm (excitation wavelength = 720 nm). However, in the presence of GSH, the 2-mercaptopyridine of **1** was replaced by nucleophilic substitution of a thiolate of GSH to form sulfur-substituted Cy7, **1-GSH**, displaying a strong fluorescence at 818 nm. However, in the presence of Hcy/Cys, **1** reacted with Hcy/Cys to form sulfur-substituted intermediates, and subsequently the amino groups of Hcy/Cys replaced the sulfur. This rearrangement furnished an amino-substituted Cy7 dyes, **1-Hcy**/**1-Cy**, exhibiting a weak fluorescence at 807 nm. In addition, a change of the GSH levels in living cells was achieved by probe **1** with confocal microscopy. Thus, **1** could be used for the discrimination of GSH over Hyc/Cys in live human cells.

## 2. Materials and Methods

### 2.1. Materials and Instrumentation

All fluorescence and UV-Vis absorption data were collected using RF-6000 (Shimadzu Corporation, Kyoto, Japan) and UV-2600 (Shimadzu Corporation) spectrophotometers, respectively. ^1^H- (500 MHz) and ^13^C-NMR (125 MHz) spectra were collected in DMSO-*d*_6_ (Cambridge Isotope Laboratories, Cambridge, MA, USA) on a Bruker spectrometer (Bruker, Billerica, MA, USA). Silica gel 60 (Merck, Darmstadt, Germany, 0.063–0.2 mm) was used for column chromatography. Analytical thin layer chromatography was performed using Merck 60 F_254_ silica gel (precoated sheets, 0.25 mm thick). All reactions were carried out under nitrogen atmosphere. All reagents including metals such as chloride salts of Na^+^, Mg^2+^, K^+^, Ca^2+^, Ni^2+^, Fe^2+^, Co^2+^, Zn^2+^, Ba^2+^, Pb^2+^, Cd^2+^, Ag^+^, Hg^2+^, Fe^3+^, Cu^2+^, and Cu^+^ ions, thiols such as cysteine (Cys), homocysteine (Hcy), glutathione (GSH), and other chemicals for synthesis, buffer solution were purchased from Aldrich (Aldrich, St. Louis, MO, USA), Alfa (Alfa, Heysham, LA3 2XY, United Kingdom), or TCI (TCI, Tokyo, Japan) and used as received. All solvents were analytical reagents. DMSO for spectroscopic experiments was HPLC reagent grade without fluorescent impurities and water was deionized water.

### 2.2. UV-Vis Absorption and Fluorescence Spectroscopic Methods

Stock solutions of synthetic compounds were prepared in DMSO. Different pH buffer solutions were prepared by using literature procedures [27]. Stock solutions of chloride salts of metal ions were prepared in water. Stock solutions of reactive oxygen species (ROS) were prepared by using literature procedures [28]. Stock solutions of thiols were prepared in 10 mM pH 7.4 HEPES buffer solution. Samples for absorption and emission measurements were contained in quartz cuvettes (4 mL volume). All data were obtained in HEPES buffer (10 mM, pH 7.4) containing 1% (*v*/*v*) of DMSO after incubation of 1 h at room temperature. Excitation was provided at 720 nm with excitation and emission slit widths being set at 10 nm.

### 2.3. Quantum Yield (Ф_F_) Measurement

Indocyanine green dye (Ф_F_ 0.13 in DMSO) was used as a reference compound for determination of the quantum yields [29]. The compound displays absorption and fluorescence with a maximum wavelength at 795 and 883 nm, respectively. Quantum yields were calculated by the comparison of the ratio between the areas for the fluorescence bands of the probe and the reference dye. The measurements were taken at the same absorbance intensity for both of the probe and the reference dye.

### 2.4. Cell Culture

MDA-MB-231, human breast carcinoma cell line, was obtained from the Korea Cell Line Bank. Cells were grown in modified Eagle’s media (MEM) (Hyclone, Logan, UT, USA) supplemented with 10% heat inactivated fetal bovine serum (Hyclone), 100 IU/mL penicillin and 100 μM/mL streptomycin (Hyclone). Cells were cultured at 37 °C in a humidified 5% CO_2_ atmosphere.

### 2.5. Cell Viability

Approximately 2 × 10^4^ cells were seeded on a 96 well microplate (SPL Life Science, Pocheon-si, Gyeonggi-do, Korea) and incubated for 24 h. After incubation, the cells were treated with water as a control and probe **1** for 6 h. To analyze the cell viability of cells in the presence of the probes, we performed using a CytoTox96^®^ Non-Radioactive Cytotoxicity Assay Kit (Promega, Madison, WI, USA) following the manufacturer’s instructions. The absorbance level was measured by a SPECTRA MAX GEMINI EM microplate reader (Molecular Devices, San Jose, CA, USA). The wavelength was set at 490 nm. Cell viability assay were performed in triplicate, and the viability (%) was expressed as a percentage of measured absorbance relative to the control cells.

### 2.6. Confocal Microscope Imaging Analysis

Approximately, cells (5 × 10^4^ cells) seeded on 35-mm glass bottom confocal dishes (SPL Life Science) and stabilized for 24 h. Cells were pretreated with the 100 μM buthionine sulfoximine (BSO) as a GSH synthesis inhibitor, and 3 mM *N*-acetyl-l-cysteine (NAC) as a ROS scavenger for 12 h and then incubated with probe **1** (10 μM in DMSO) at 37 °C in 5% CO_2_ for 2 h. Fluorescence microscope images of probe **1**-labeded cells were obtained with a confocal laser scanning microscope (CLSM, Carl-Zeiss LSM 700 Exiciter, Heidelberg, Germany) by excited at 633 nm with a He/Ne optics laser source and emission was collected by a 640–690 nm band pass filter.

### 2.7. Computational Study

The geometry and associated energies of the tetrahedral intermediates of probe **1** with GSH and Cys were calculated using the ωB97XD functional [30] at the 6-31+G(d) level of theory [31,32,33,34] with the default IEFPCM variant of the PCM model of water [35] using the Gaussian 16 software suite [36]. The structural optimizations and frontier orbital calculations of molecular orbitals of **1-H^+^**, **Cy7-SMe** and **Cy7-NHMe** were performed using the PBE0 functional [37] at the 6-311G(2d,p) level of theory [31,32,34] using the default IEFPCM variant of the PCM model of water. Input files and structure and molecular orbital visualizations were generated using Gabedit 2.5.0 [38].

### 2.8. Synthesis

**Scheme 2:**
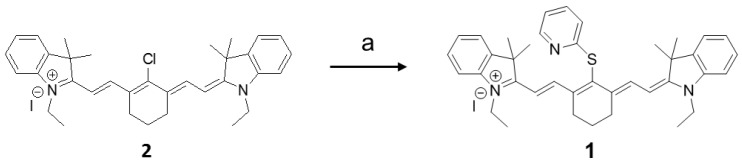
Synthetic route for **1**. *Reaction conditions*: (a) 2-mercaptopyridine, NaH, DMF, 0 °C, 2 h.

Probe **1** was prepared by the synthetic route depicted in Scheme 2. Briefly, compound **2** was reacted with 2-mercaptopyridine in the presence of NaH as a base in DMF, which produced **1** in 70% yield. The chemical structure of **1** was identified by ^1^H and ^13^C NMR spectroscopy, as well as ESI-MS spectrometry (Figures S10–S12).

#### 2.8.1. Synthesis of **2**

Cy7 dye (**2**) was prepared according to the previously published procedure [39].

#### 2.8.2. Synthesis of **1**

To a single necked round bottom flask under an inert atmosphere, NaH (11 mg, 48 mmol) was added to a cold solution of DMF (10 mL) and stirred for 15 min. 2-Mercaptopyridine (53 mg, 0.48 mmol) was added to the mixture dropwise and stirring was continued for additional 30 min. Finally, DMF (5 mL) solution of **2** (200 mg, 0.32 mmol) was added dropwise and further stirred at 0 °C for additional 1 h. After completion, the reaction was quenched with brine, extracted with dichloromethane (DCM) (3 × 50 mL). The organic extract was dried over anhydrous sodium sulfate, filtered, concentrated and purified by a silica gel column chromatography (5–10% MeOH in DCM) to obtain **1** (160 mg, 70% yield) as a blue powder. ^1^H-NMR (DMSO-*d*_6_, 28 °C): δ 1.37 (t, 6H, *J* = 7.2 Hz), 1.46 (brs, 12H), 2.06 (s, 2H), 2.82 (m, 4H), 4.18 (q, 4H, *J* = 7.2 Hz), 6.32 (*d*, 2H, *J* = 14.2 Hz), 7.11–7.13 (m, 2H), 7.15–7.18 (m, 8H), 7.64–7.67 (m, 1H), 8.38-8.39 (m, 1H), 8.78 (m, 2H, *J* = 14.2 Hz) ppm. ^13^C NMR (DMSO-*d*_6_, 28 °C): δ 11.2, 20.7, 25.9, 26.6, 38.9, 49.1, 101.0, 110.6, 120.7, 122.1, 125.1, 128.5, 128.6, 133.4, 138.1, 141.3, 141.7, 145.8, 145.8, 149.2, 172.3 ppm. ESI-MS: *m*/*z* C_39_H_44_N_3_S^+^ calcd. 586.33, found 586.30 [M]^+^.

## 3. Results and Discussion

### 3.1. Photophysical Study of **1** for the Detection of GSH over Cys/Hcy

**Figure 1:**
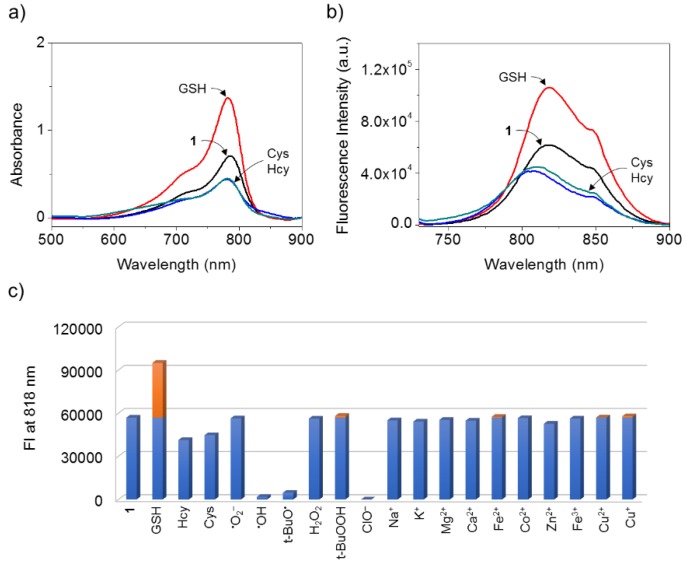
(**a**) UV-Vis absorption and (**b**) fluorescence spectra of **1** (10 μM) in presence GSH, Hcy and Cys (10 mM, respectively). (**c**) Relative fluorescence intensity at 810 nm of **1** to thiols, ROS, and metal ions. All data were obtained in HEPES buffer (10 mM, pH 7.4) containing 1% (*v*/*v*) of DMSO after incubation of 1 h at room temperature. The excitation wavelength was affected at 720 nm.

We investigated UV-Vis absorption and fluorescence changes of **1** to various metal ions, anions, reactive oxygen species (ROS), and thiols in HEPES buffer (10 mM, pH 7.4) containing 1% (*v*/*v*) of DMSO. As shown in Figure 1a,b, probe **1** exhibited absorption and fluorescence bands at 790 and 818 nm, respectively. However, in the presence of GSH, the absorption at 790 nm was greatly enhanced with increased fluorescence intensity at 818 nm. In contrast, in presence of Hcy/Cys, the absorption of **1** at 790 nm was slightly diminished, while the fluorescence intensity distinctively decreased along with a shift of wavelength maximum from 818 to 807 nm. The quantum yield (Φ_F_) of **1** was measured in the absence and presence of GSH, Cys, and Hcy, resulted in 0.006, 0.016, 0.003, and 0.011 for **1**, **1-GSH**, **1-Cys**, and **1-Hcy**, respectively.

We next tested the fluorescence response of **1** towards other biological analytes, including ROS (e.g., ^•^O_2_^−^, ^•^OH, ^t^BuO^•^, H_2_O_2_, ^t^BuOOH, ClO^−^) and metal ions (e.g., Na^+^, K^+^, Mg^2+^, Ca^2+^, Fe^2+^, Co^2+^, Zn^2+^, Fe^3+^, Cu^2+^, Cu^+^) (see also Figures S1 and S2). The fluorescent increase of **1** observed for GSH was not observed upon the addition of any of the potential interferants tested (Figure 1c). In addition, probe **1** showed the fluorescence increase for GSH even in the presence of other analytes except for iron ions and ClO^−^ (Figure S3). These results indicate that **1** can provide a highly selective fluorescence response at 818 nm to GSH over Hcy/Cys and other potential interferants.

**Figure 2:**
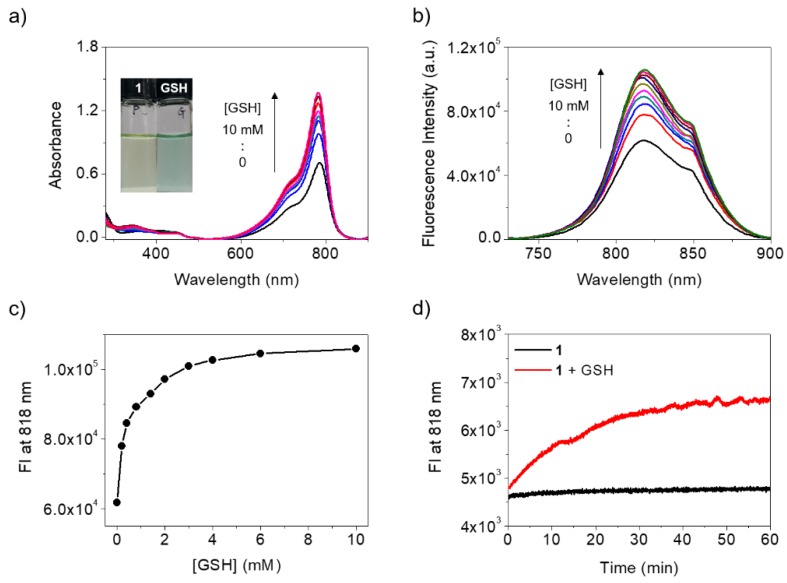
(**a**) UV-Vis absorption and (**b**) fluorescence spectra of **1** (10 μM) in presence of different concentrations of GSH (0–10 mM); (**c**) plot of fluorescence intensity at 818 nm of **1** vs. [GSH]; (**d**) time-dependent fluorescence change of **1** (10 μM) to 10 mM of GSH. All data was obtained in HEPES buffer (10 mM, pH 7.4) containing 1% (*v*/*v*) of DMSO after incubation of 1 h at room temperature. The excitation wavelength was affected at 720 nm.

To get insight into the photophysical changes of **1** to GSH, probe **1** was treated with different concentrations of GSH. As the concentration of GSH increased, **1** exhibited a gradual increase in the absorption at 790 nm as well as a visual color change from light green to deep green (Figure 2a, inset). On the other hand, the fluorescence intensity of probe **1** at 818 nm was gradually increased and saturated at 10 mM of GSH as the increase of GSH concentration (Figure 2b,c). These changes depending on the GSH concentration might be due to a formation of **1-GSH**, as depicted in Scheme 1. In addition, the change in fluorescence intensity (FI) at 818 nm was plotted as [GSH] concentrations of 0–600 µM (Figure S4). From this, the limit of detection (LOD, 3σ/s) [40] of **1** for GSH was assessed as 97 µM, which is in the range of biological concentration of GSH.

By contrast, upon increasing concentrations of Cys or Hcy, probe **1** exhibited a significant decrease in the absorption at 790 nm and showed a fluorescence decrease as well as the fluorescence wavelength shift from 818 to 807 nm (Figures S5 and S6). This change might result from the formation of the amino-substituted Cy7 dyes, **1-Hcy**/**1-Cys**, as suggested in Scheme 1, unlike GSH.

Moreover, fluorescence changes of **1** in the presence of GSH were monitored over the course of time. Upon adding 10 mM of GSH to a solution of **1**, the fluorescence intensity at 818 nm was increased and saturated within 1 h (red line in Figure 2d). By contrast, in the absence of GSH, **1** showed a negligible fluorescence change (black line in Figure 2d). On the other hand, in the presence of Cys, the fluorescence intensity of **1** at 818 nm was gradually decreased in 60 min (Figure S7). In the case of Hcy, **1** showed a rapid decrease in the fluorescence intensity at 818 nm within 4 min, followed by the gradual enhancement in fluorescence up to 60 min. The two-phase fluorescence change of **1** to Hcy is probably due to the formation of a sulfur-substituted Cy7 intermediate followed by the rearrangement of the resulting amino-substituted Cy7, **1-Hcy**.

In addition, the effect of the pH on probe **1**’s ability for GSH detection was also tested. In the presence of 10 mM of GSH, **1** showed a distinct fluorescence increase at 818 nm in the pH range of 6–12 (Figure S8). It is noteworthy that the pH range of 6–12 is biologically relevant. Along with the results obtained from Figure 1 and Figure 2, this indicated that probe **1** could provide a fluorescence increase at 818 nm towards GSH over Cys/Hcy in physiological conditions. We could think that it is ambiguous to judge whether the non-fluorescent cellular image of **1** is due to the absence of GSH or the presence of Cys/Hcy. However, in consideration of the physiological abundance of GSH compared to Cys/Hcy, the interference from Cys/Hcy would be insignificant. Thus, we believed that **1** could be used as a promising NIR fluorescent probe for the selective detection of GSH in human cells.

**Figure 3:**
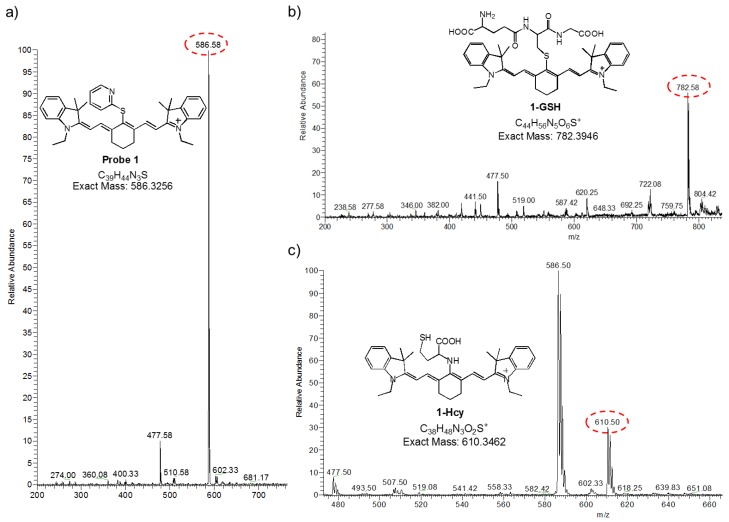
ESI-MS spectra of **1** (**a**) in the absence and presence of (**b**) GSH or (**c**) Hcy.

To gain insights into the reaction between probe **1** with GSH, Cys, and Hcy, we next performed mass analyses using **1** in the presence of GSH, Cys, and Hcy. In Figure 3, probe **1** showed a mass peak at 586 *m*/*z*. However, in the presence of GSH, **1** exhibited a mass peak at 782 *m*/*z* corresponding to the formation of **1-GSH**, while in the case of Hcy a mass peak at 610 *m*/*z* for **1-Hcy** appeared. For Cys, the resulting intermediate **1-Cys** did not seem to be ionized. However, as revealed in Figure 1, probe **1** showed quite different absorption and fluorescence changes for GSH and Cys/Hcy. Noticeably, **1** displayed the fluorescence centered at 818 nm in the absence and presence of GSH, whereas the fluorescence maximum wavelength was changed to 807 nm for Cys/Hcy. This can be derived from the formations of sulfur-substituted Cy7 for GSH (**1-GSH**) and amino-substituted Cy7 for Cys/Hcy (**1-Cys**/**1-Hcy**). Collectively, all photophysical changes and mass spectroscopic results supported the sensing mechanism of **1** for the selective detection of GSH over Cys/Hcy, as proposed in Scheme 1.

### 3.2. Theoretical Study of Probe **1** and the Interaction with GSH and Cys

**Figure 4:**
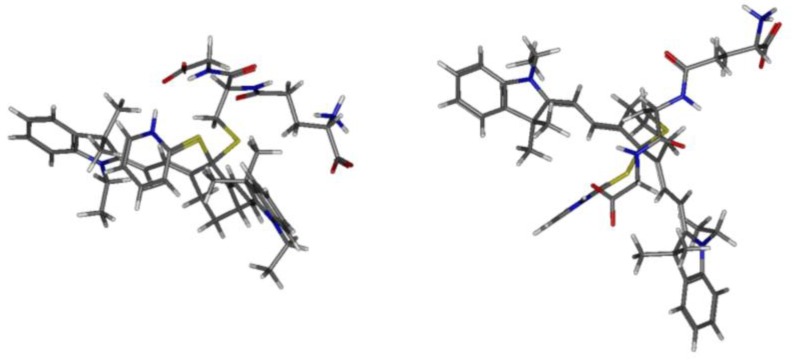
Optimized structure of the tetrahedral intermediate resulting from the nucleophilic addition of GSH to **1** (PCM-ωB97XD/6-31+G(d)). **Left**: side view, **Right**: top view. See Movie 1 for an animated version of the structure.

The selectivity for GSH over other bio-thiols could be rationalized by DFT studies. Theoretical studies regarding the interaction between GSH and **1** were carried out using the dispersion-corrected range-separated ωB97XD functional, which was previously demonstrated to most accurately describe the sulfur bond distances in intermediates of thiol-Michael additions [41], at the 6-31+G(d) level of theory in water. As can be seen in Figure 4 and Movie 1, GSH stabilizes the formation of the tetrahedral intermediate by the formation of a hydrogen bond between the glycine carboxylic acid and the pyridine. The formation of the hydrogen bond with the pyridine ring results in preorganization of GSH’s sulfur atom near the Cy7 scaffold’s reactive center, thus lowering the transition energy of the addition-elimination reaction, resulting in an accelerated reaction.

**Figure 5:**
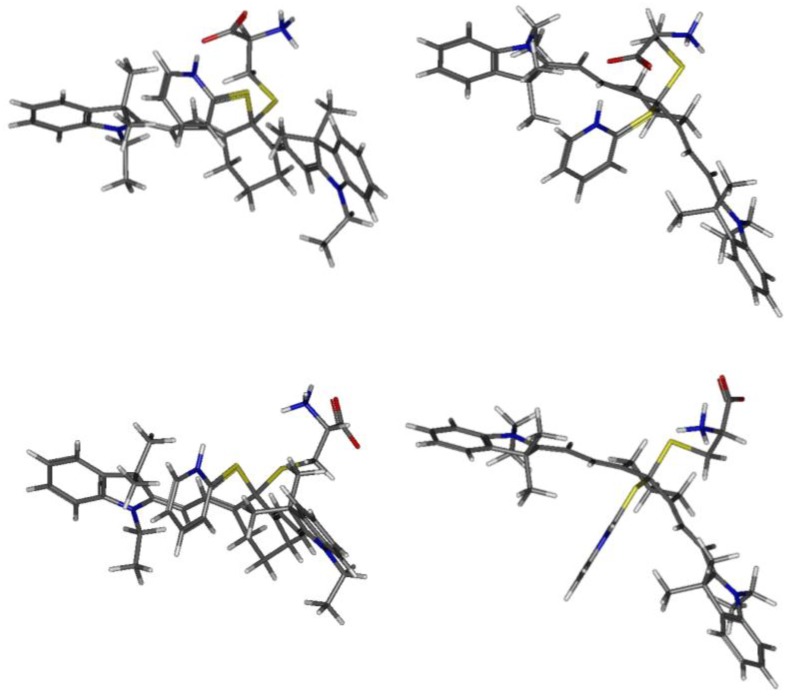
Optimized structure of the tetrahedral intermediate resulting from the nucleophilic addition of Cys to **1** (PCM-ωB97XD/6-31+G(d)), **Top**: cyclic intermediate, **Bottom**: acyclic intermediate, **Left**: side view, **Right**: top view. See Movies 2 and 3 for an animated version of both structures.

Cysteine, on the other hand, would require an energetically unfavorable 10-membered ring in a similar preorganized tetrahedral state intermediate. Calculations predict only a small difference (1.02 kCal·mol^−1^) between a cyclic and acyclic tetrahedral intermediate at the ωB97XD/6-31+G(d) level of theory in water (Figure 5 and Movies 2–3), with the acyclic tetrahedral intermediate possessing the lowest energy. This small energy difference signifies the absence of a clear bias for either the cyclic or acyclic tetrahedral intermediate. As the addition step in the reaction of **1** with either GSH or Cys is the rate determining step (Scheme 1), the observed half-life of the reaction clearly shows the importance of the preorganization in the reaction kinetics (16 min vs. 27 min, for GSH and Cys, respectively, see Figure 2 and Figure S7). Hcy, by contrast, exhibits a complex reaction kinetics behavior that was not further elucidated via calculations (Figure S7).

### 3.3. Frontier Molecular Orbitals of N- and S-Substituted Cy7 Compounds

The structures of **1-H^+^**, **Cy7-SMe** and **Cy7-NHMe**, as a model structure for the *S*- and *N*-substituted Cy7 dyes, respectively were optimized at the PBE0/6-311G(2d,p) level of theory using the PCM model of water. The optimized geometries and associated frontier orbitals are depicted in Figure S13.

### 3.4. Fluorescence Imaging of the Cellular GSH Using Probe **1** in Live Human Cells

**Figure 6:**
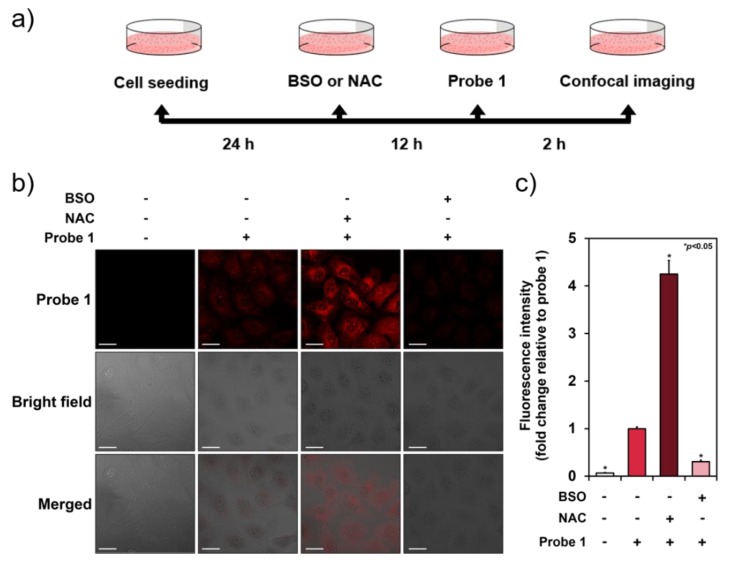
Confocal laser scanning microscopy images of **1** in MDA-MB-231 cells. (**a**) Preparation of cells for the confocal imaging using probe **1**, buthionine sulfoximine (BSO), and *N*-acetyl-l-cysteine (NAC); (**b**) cells were pretreated with BSO (100 µM; GSH inhibitor) or NAC (3 mM; ROS scavenger) for 12 h and then incubated with **1** (10 µM) for 2 h. Scale bar = 20 μm; (**c**) the relative fluorescence intensity (fold change) of probe **1** was presented. The mean values of each group were quantified in 40 cells per treatment condition, after setting a common threshold for all images using Image J. Data are represented as fold change vs. the indicated reagents. The deep red bar indicated a fluorescence increase after NAC treatment, whereas pink bar represented a fluorescence decrease after BSO treatment. The percent fold changes are all significant (*p < 0.05) based on a *t*-test.

We next tested the ability of probe **1** to image the cellular GSH by using confocal microscopy and the results are shown in Figure 6. Before applying **1** to bioimaging, cytotoxic effect of probe **1** was estimated in MDA-MB-231 cells by using CytoTox96^®^ Non-Radioactive Cytotoxicity Assay. The cell viability assays showed that up to 30 μM, **1** exhibited a very low cytotoxicity (<6%) in cells (Figure S9). This result indicated that probe **1** has a potential for further biosensing applications. The MDA-MB-231 cells were incubated with probe **1** after the pretreatments of *N*-acetyl-l-cysteine (NAC) [42] as a ROS scavenger and buthionine sulfoximine (BSO) [43] as a GSH inhibitor (Figure 6a). Probe **1** displayed a very weak red fluorescence in intact cells without NAC and BSO (Figure 6b). However, probe **1** showed a strong red fluorescence in the NAC-treated cells, presumably due to the increased level of antioxidant GSH by the ROS scavenging. In contrast, in the case of BSO-treated cells, a significant decrease in the fluorescence intensity was observed, indicating the decreased GSH level. The relative fluorescence intensity of the images was also quantified in Figure 6c. Hence, we demonstrated that probe **1** could selectively react with GSH to give a fluorescence increase in the live human cells.

## 4. Conclusions

We presented a highly selective NIR fluorescent probe (**1**) that can detect GSH selectively over Cys/Hcy under physiological conditions. We propose that in the presence of GSH and Hcy/Cys, the 2-mercaptopyridine of **1** is replaced by the thiolate of GSH to form the sulfur-substituted Cy7, **1-GSH**, displaying a strong fluorescence at 818 nm. However, for Hcy/Cys, **1** reacted with Hcy/Cys to form sulfur-substituted intermediates, and subsequently in a rearrangement reaction the amino groups of Hcy/Cys replaced the sulfur to give rise to the amino-substituted Cy7 dyes, **1-Hcy**/**1-Cy**, exhibiting a weak fluorescence at 807 nm. The sensing mechanism was thoroughly identified by photophysical studies, mass analyses, and computational calculations. In addition, we showed that the fluorescence increase of probe **1** was highly selective for GSH over Hcy, Cys, and other potential interferants such as biologically abundant ROS and metal ions. Moreover, we identified the ability of probe **1** for the selective detection of GSH in MDA-MB-231 cells using *N*-acetyl-l-cysteine (NAC; GSH inducer) or buthionine sulfoximine (BSO; GSH inhibitor). We envision that probe **1** will be a promising tool for imaging and monitoring of the GSH levels in living cells and will stimulate the design of new types of biologically relevant probes in the near future.

## Supplementary Materials

The following are available online at http://www.mdpi.com/1424-8220/18/9/2897/s1, Figure S1: UV-Vis absorption and fluorescence spectra of **1** (10 μM) in presence of reactive oxygen species such as ^•^O_2_^−^, ^•^OH, t-BuO^•^, H_2_O_2_, t-BuOOH, and ClO^−^ (1 mM, respectively). Figure S2: UV-Vis absorption and fluorescence spectra of **1** (10 μM) in presence of various anions such as various metal ions Na^+^, K^+^, Mg^2+^, Ca^2+^, Fe^2+^, Co^2+^, Zn^2+^, Fe^3+^, Cu^2+^, and Cu^+^ (1 mM, respectively). Figure S3: Absorption and fluorescence spectra of **1** (10 μM) in presence of different concentrations of Cys (0–3 mM). Figure S4: Absorption and fluorescence spectra of **1** (10 μM) in presence of different concentrations of Hcy (0–1 mM). Figure S5: Time-dependent fluorescence responses of **1** (10 μM) toward (a) Cys and (b) Hcy (10 mM) in HEPES buffer (0.01 M, pH 7.4) containing 1% (*v*/*v*) of DMSO. Figure S6: The pH effect on fluorescence change of **1** (10 μM) in absence (black line) and presence (red line) of GSH (10 mM). Figure S7: ^1^H NMR spectrum of **1** in DMSO-*d*_6_. Figure S8: ^13^C NMR spectrum of **1** in DMSO-*d*_6_. Figure S9: ESI-MS spectrum of **1**. Figure S10: Isodensity surface (0.03 e.bohr^−3^) of the HOMO (Left) and LUMO (Right) orbitals of a) **Cy7-NHMe**, b) **Cy7-SMe** and c) **1-H^+^** at the PCM-PBE0/6-311G(2d,p) level of theory. Table S1: Coordinates of optimized structures.
