# Supplementary material for: A Fluorescent Cy7-Mercaptopyridine for the Selective Detection of Glutathione over Homocysteine and Cysteine
Source: Sensors (Basel). 2018 Sep 1;18(9):2897. doi: 10.3390/s18092897 (PMC6164685; doi:10.3390/s18092897)
Supplement: Supplementary file 1 [file sensors-18-02897-s001.zip › Supplementary Information.pdf]

Supporting Information

A Fluorescent Cy7-Mercaptopyridine for the Selective Detection of Glutathione over Homocysteine and Cysteine

Shin A Yoon [1,†], Wantae Kim [2,†], Amit Sharma [3,†], Peter Verwilst [3], Miae Won [3] and Min Hee Lee [1,*]

1 Department of Chemistry, Sookmyung Women's University, Seoul 04310, Korea; dbstlsdk23@sookmyung.ac.kr
2 School of Chemical and Biological Engineering, Seoul National University, Seoul 151-742, Korea; wtkim223@naver.com
3 Department of Chemistry, Korea University, Seoul 02841, Korea; amitorg83@gmail.com (A.S.); peterverwilst@hotmail.com (P.V.); miaewon@korea.ac.kr (M.W.)
* Correspondence: minheelee@sookmyung.ac.kr; Tel.: +82-02-2077-7815
† These authors contributed equally.

Contents

1. Supplementary data

2. ^{1}H- ^{13}C-NMR, and ESI-MS spectra of 1

3. Computational data

1. Supplementary data

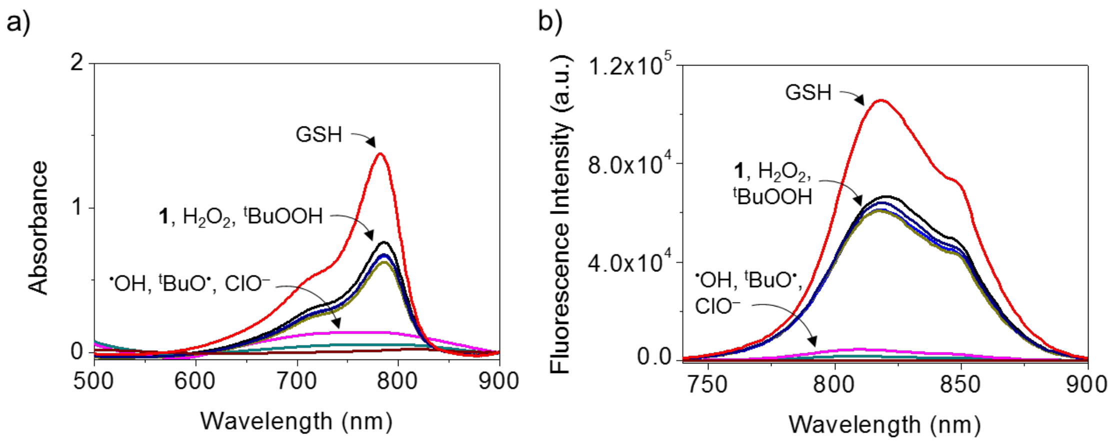

Figure S1. (a) UV-Vis absorption and (b) fluorescence spectra of **1** (10 μM) in presence of reactive oxygen species such as $^{\bullet}O_2^{-}$, $^{\bullet}OH$, t-BuO$^{\bullet}$, H_2O_2, *t*-BuOOH, and ClO^{-} (1 mM, respectively). All data was obtained in HEPES buffer (10 mM, pH 7.4) containing 1% (*v*/*v*) of DMSO after incubation of 1 h at room temperature. The excitation wavelength was affected at 720 nm.

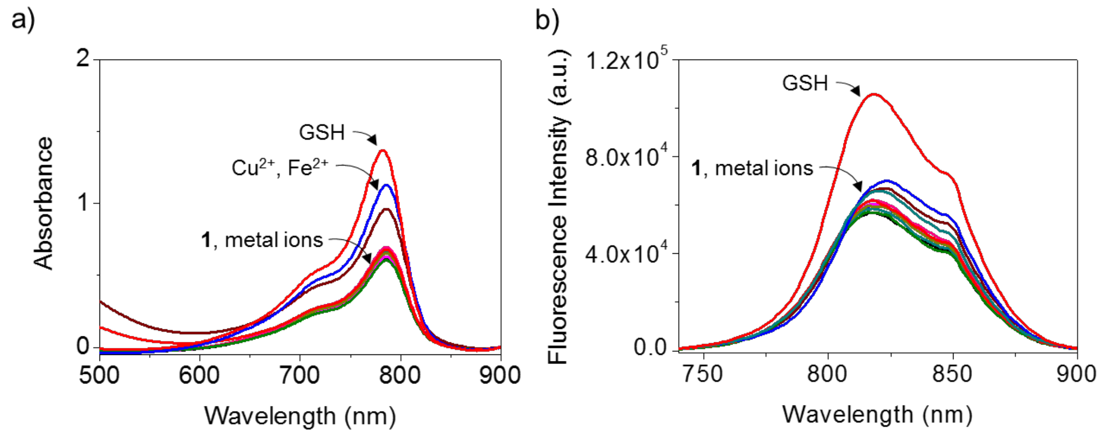

Figure S2. (a) UV-Vis absorption and (b) fluorescence spectra of **1** (10 μM) in presence of various anions such as various metal ions Na^{+}, K^{+}, Mg^{2+}, Ca^{2+}, Fe^{2+}, Co^{2+}, Zn^{2+}, Fe^{3+}, Cu^{2+}, and Cu^{+} (1 mM, respectively). All data was obtained in HEPES buffer (10 mM, pH 7.4) containing 1% (*v*/*v*) of DMSO after incubation of 1 h at room temperature. The excitation wavelength was affected at 720 nm.

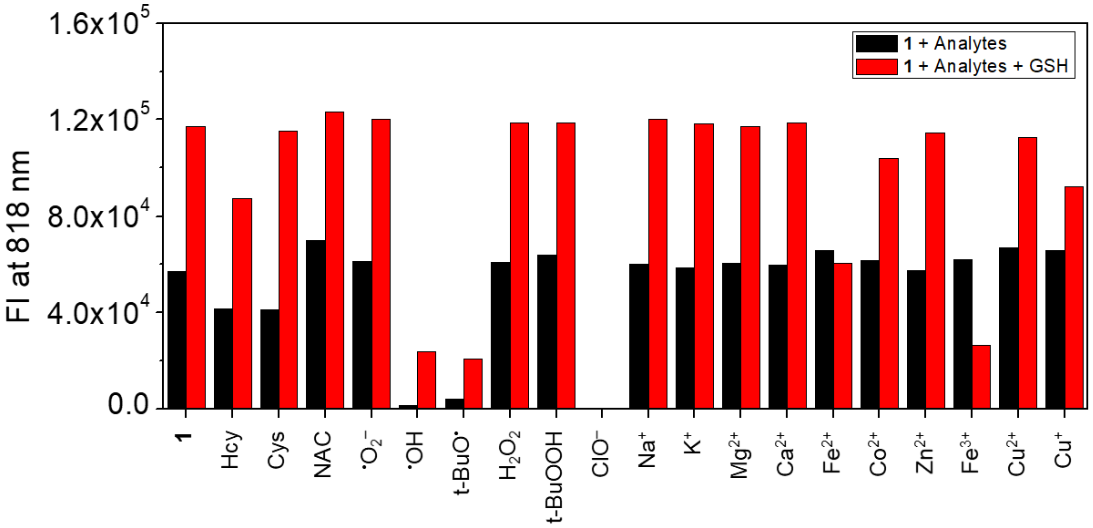

Figure S3. Relative fluorescence intensity at 818 nm of **1** (10 μM) in the absence and presence of GSH with other analytes such as thiols (10 mM), ROS (1 mM), and metals (1 mM). Black bars indicate fluorescence intensity of **1** in the absence and presence of various analytes. Red bars indicate fluorescence intensity of **1** towards GSH in the presence of other analytes. All data were obtained in HEPES buffer (10 mM, pH 7.4) containing 1% (*v*/*v*) of DMSO after incubation of 1 h at room temperature. The excitation wavelength was affected at 720 nm.

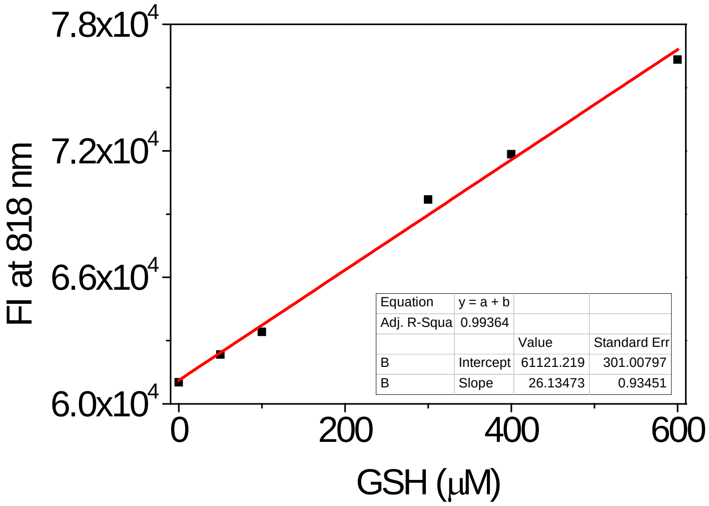

Figure S4. Plot of fluorescence intensity at 818 nm of **1** *vs.* [GSH] in the range of 0-600 μM.

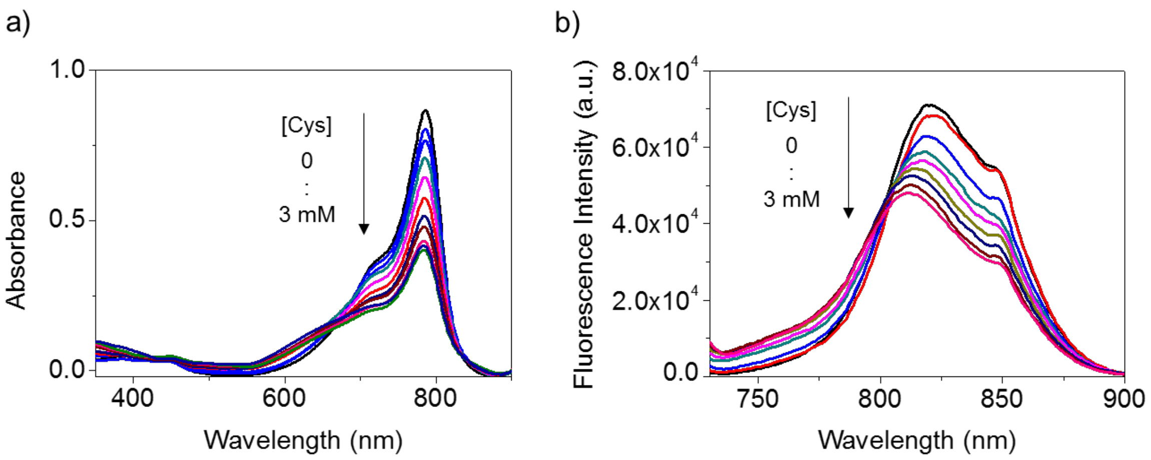

Figure S5. (a) Absorption and (b) fluorescence spectra of **1** (10 μM) in presence of different concentrations of Cys (0-3 mM). All data was obtained in HEPES buffer (10 mM, pH 7.4) containing 1% (*v*/*v*) of DMSO after incubation of 1 h at room temperature. The excitation wavelength was affected at 720 nm.

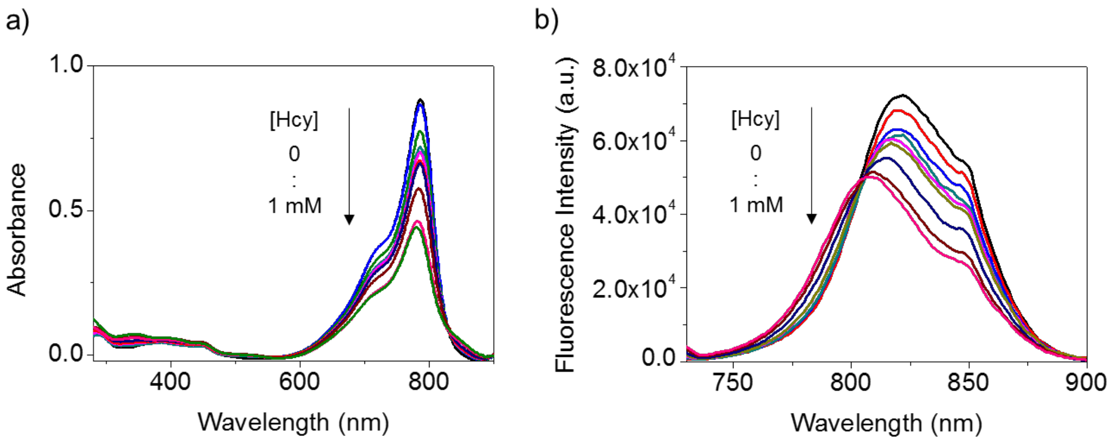

Figure S6. (a) Absorption and (b) fluorescence spectra of **1** (10 μM) in presence of different concentrations of Hcy (0-1 mM). All data was obtained in HEPES buffer (10 mM, pH 7.4) containing 1% (*v*/*v*) of DMSO after incubation of 1 h at room temperature. The excitation wavelength was affected at 720 nm.

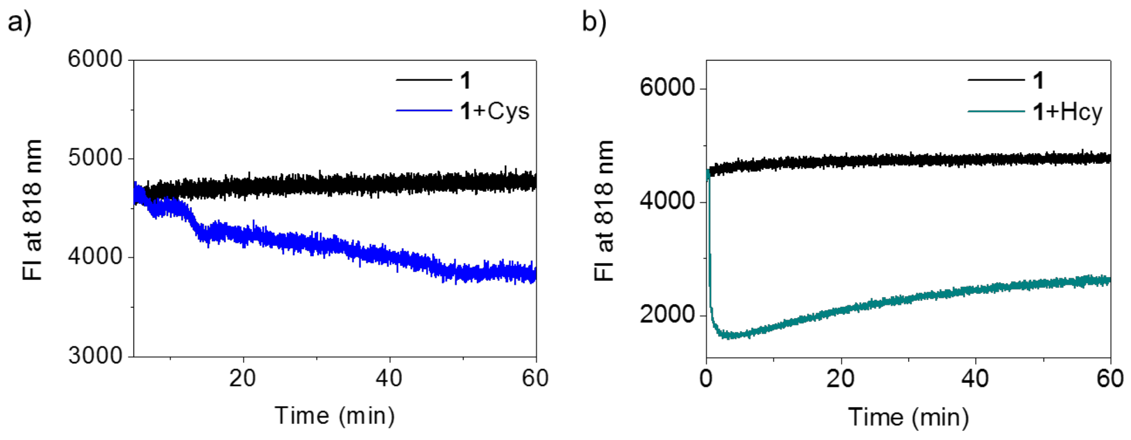

Figure S7. Time-dependent fluorescence responses of **1** (10 μM) toward (a) Cys and (b) Hcy (10 mM) in HEPES buffer (0.01 M, pH 7.4) containing 1% (*v*/*v*) of DMSO.

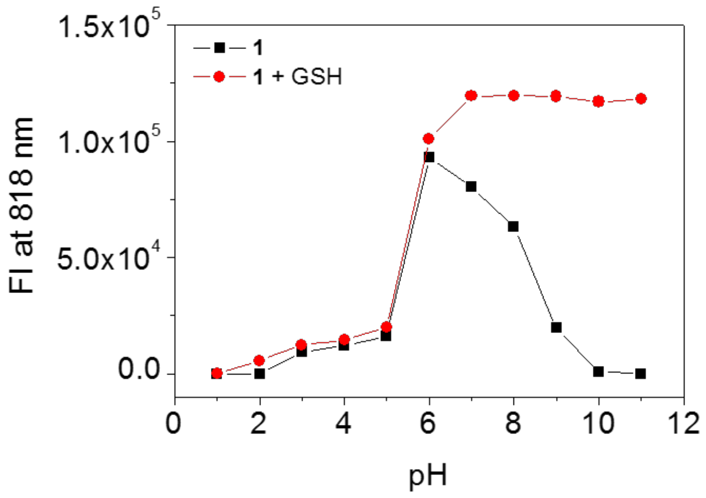

Figure S8. The pH effect on fluorescence change of **1** (10 μM) in absence (black line) and presence (red line) of GSH (10 mM). All data was obtained in HEPES buffer (10 mM, pH 7.4) containing 1% (*v*/*v*) of DMSO after incubation of 1 h at room temperature. The excitation wavelength was affected at 720 nm.

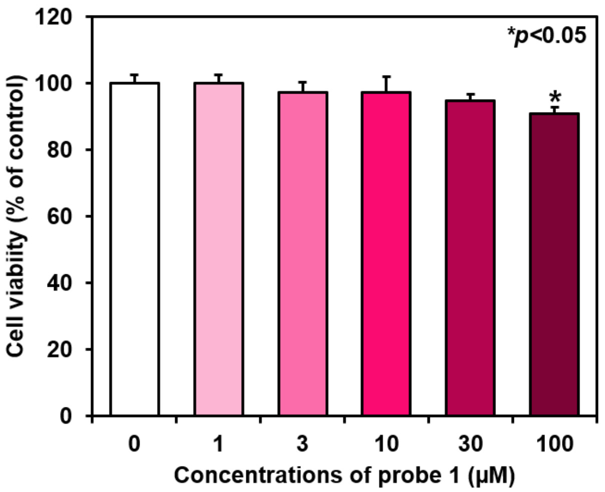

Figure S9. Cell viability of probe **1**. Cells were incubated with 0, 1, 3,10, 30 and 100 μM of probe **1** for 6 h in MDA-MB-231 cells. The values represent mean ± SE of three independent experiments performed in triplicate

2. ¹H, ¹³C NMR and ESI-MS spectra of 1

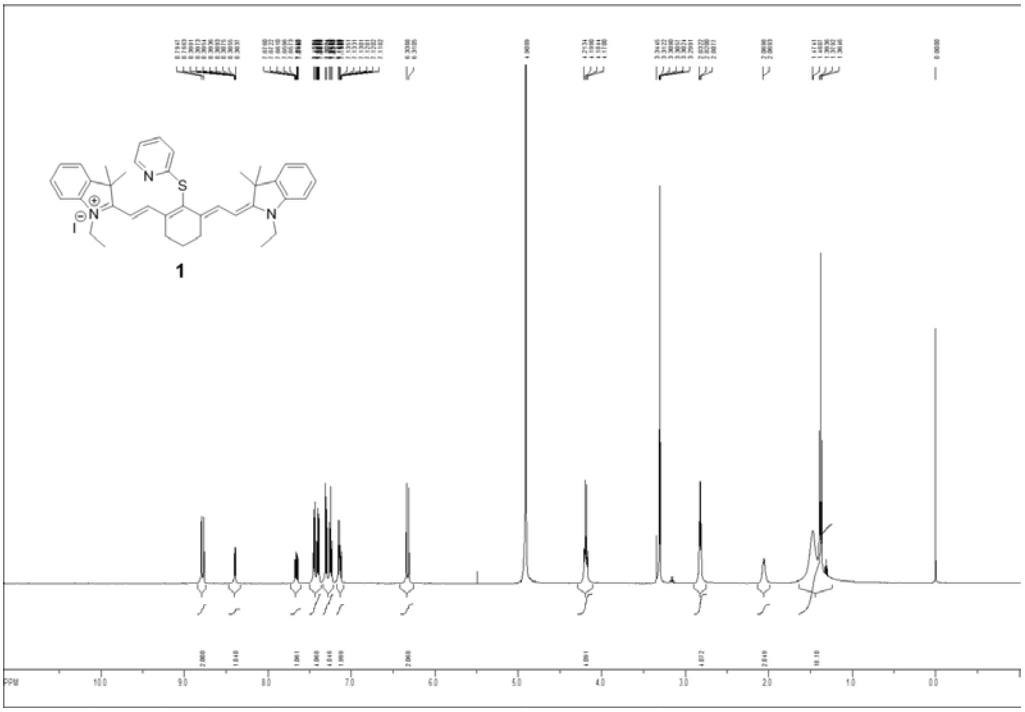

Figure S10. ¹H-NMR spectrum of **1** in DMSO-d_6.

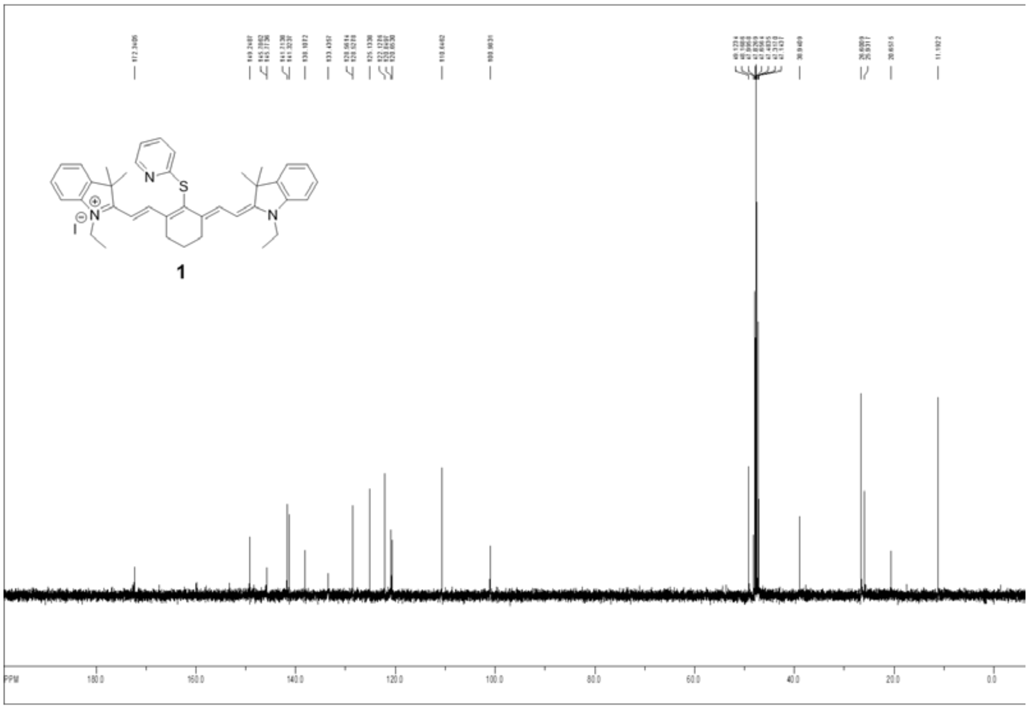

Figure S11. ^{13}C-NMR spectrum of **1** in DMSO-d_6.

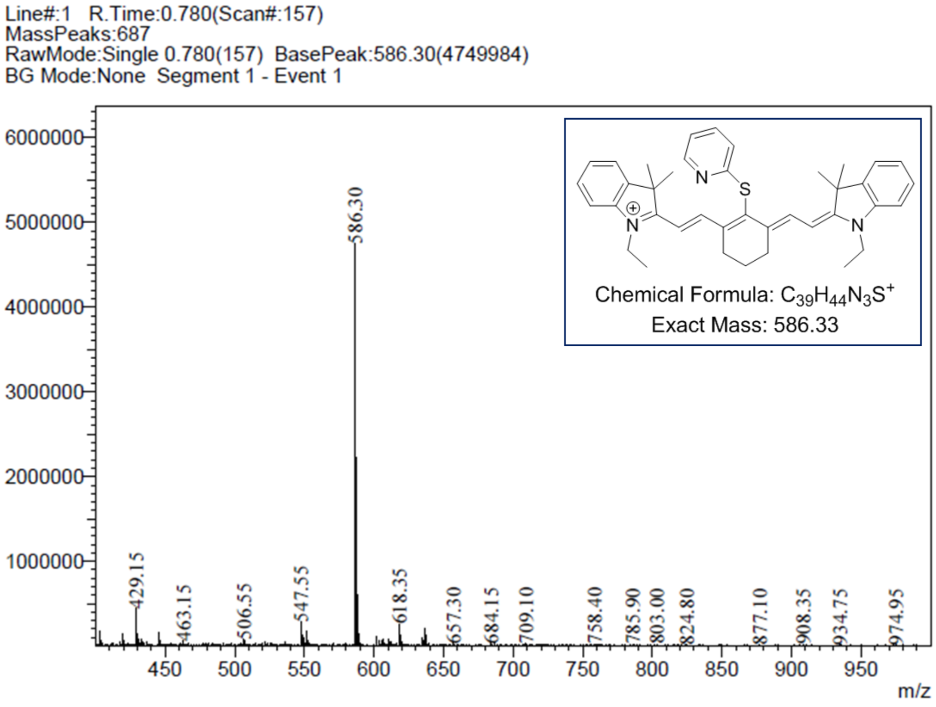

Figure S12. ESI-MS spectrum of **1**.

3. Computational data

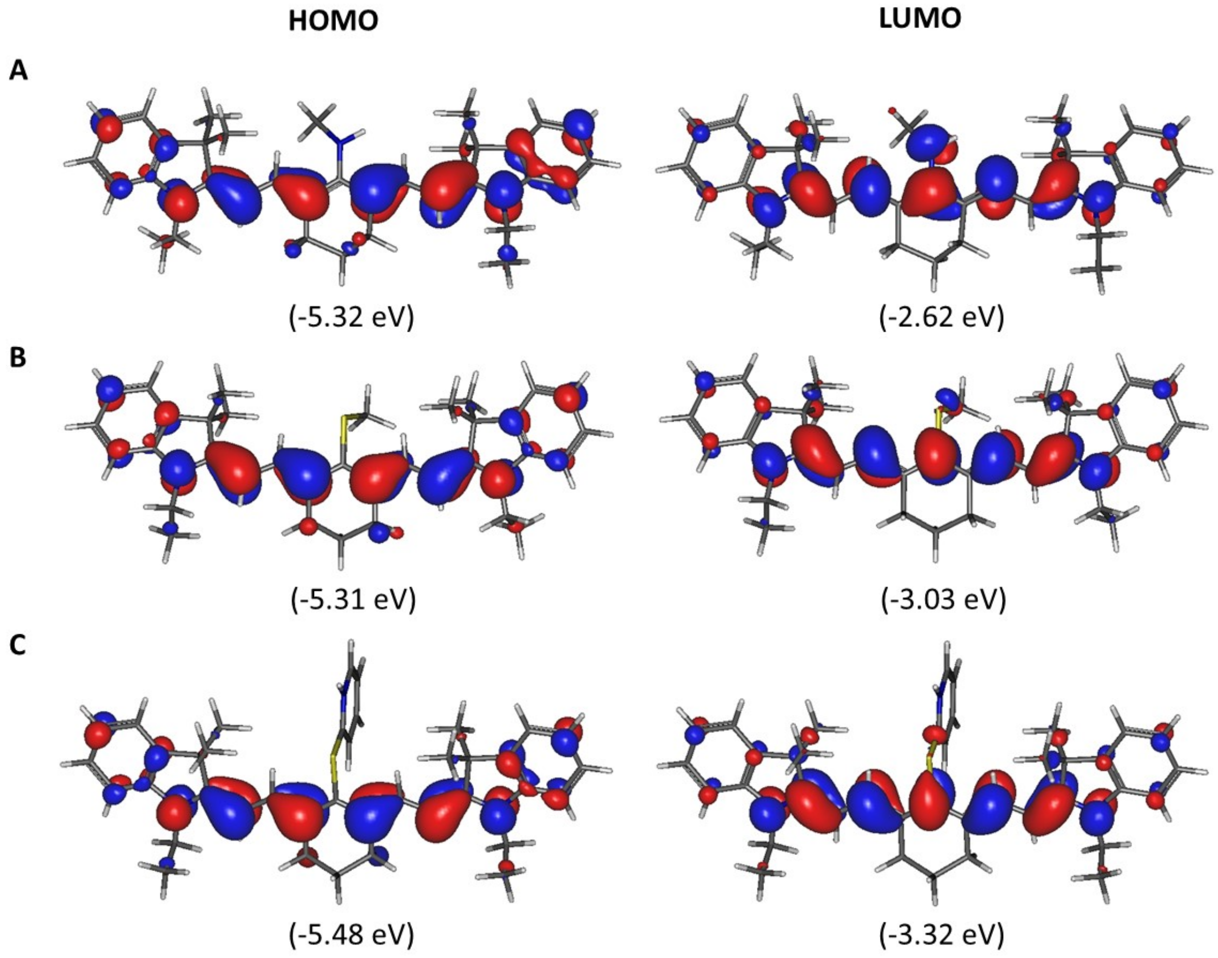

Figure S13. Isodensity surface (0.03 e.bohr^{-3}) of the HOMO (Left) and LUMO (Right) orbitals of a) **Cy7-NHMe**, b) **Cy7-SMe** and c) **1-H$^+$** at the PCM-PBE0/6-311G(2d,p) level of theory. The associated orbital energies are shown between brackets in eV.

Table S1. Coordinates of optimized structures

1-GSH E(RωB97XD) = -3478.927477 Ha

Center Number	Atomic Number	Coordinates (Angstroms) X	Y	Z
1	1	0.483239	-0.429076	-3.761788
2	1	5.320828	-1.085730	-4.004515
3	1	6.979636	-1.716872	-3.971111
4	1	0.289969	1.205775	-4.406272
5	6	6.377155	-0.802676	-3.955926
6	1	4.448214	-3.452245	-0.620372
7	1	-1.810459	0.420070	-3.351126
8	6	0.329444	0.621040	-3.480535

9	6	-0.987529	0.763004	-2.721307
10	1	3.247457	-2.632360	0.392895
11	6	4.303444	-2.901286	0.314307
12	1	4.538522	-3.570515	1.148811
13	1	4.133871	0.375368	-2.338972
14	6	6.666832	0.010712	-2.695788
15	6	1.555090	0.348943	-1.302243
16	1	-1.165517	1.830344	-2.519123
17	6	4.008565	-0.171753	-1.411122
18	1	9.094300	-1.317632	-1.984280
19	6	2.692996	-0.187875	-0.811527
20	7	6.359734	-0.711779	-1.476469
21	6	0.233787	0.277426	-0.533533
22	6	5.103962	-0.788958	-0.892816
23	6	8.618053	-1.751411	-1.110722
24	1	7.722062	0.295476	-2.657847
25	6	7.282193	-1.511149	-0.803341
26	1	2.618609	-0.687805	0.145541
27	1	10.385267	-2.792005	-0.473664
28	6	9.340699	-2.589578	-0.254059
29	6	5.217853	-1.663767	0.370511
30	6	6.671613	-2.094253	0.308585
31	16	0.320810	-0.738257	1.007043
32	6	8.745414	-3.168713	0.865696
33	6	7.395149	-2.919903	1.149785
34	1	9.325596	-3.816815	1.515573
35	6	4.967070	-0.849084	1.654799
36	1	3.929675	-0.510049	1.727021
37	1	5.180372	-1.470673	2.531159
38	1	5.619324	0.029348	1.689745
39	6	0.283786	-2.432433	0.530907
40	7	-0.115486	-3.269819	1.514596
41	6	-0.134233	-4.607301	1.373555
42	6	0.237106	-5.184926	0.179218
43	6	0.623255	-4.347612	-0.870773
44	6	0.653831	-2.970214	-0.699926
45	1	-0.468936	-5.168838	2.236757
46	1	0.215029	-6.261543	0.068739
47	1	0.912080	-4.772582	-1.826198
48	1	6.618378	-0.215189	-4.847515
49	1	-3.338765	-4.372777	-1.211652
50	1	2.430610	0.987386	-3.157894
51	6	1.491075	1.098390	-2.612688
52	1	-2.213922	-3.386514	-0.270677
53	6	-3.211512	-3.831210	-0.269039
54	1	-3.268788	-4.551474	0.554133
55	1	-3.447499	-0.373089	-2.601972
56	6	-0.978341	0.001648	-1.415607
57	1	1.365466	2.173551	-2.418239
58	6	-3.277429	-0.953266	-1.702022
59	1	-8.092454	-2.826336	-2.615174
60	6	-2.018725	-0.760681	-1.013024
61	7	-5.507687	-1.848340	-1.932179
62	6	-4.282923	-1.769020	-1.286598
63	1	6.098351	0.945794	-2.693563
64	6	-7.589185	-3.244439	-1.749180

65	6	-6.320368	-2.825920	-1.360030
66	1	-9.196761	-4.585387	-1.270709
67	6	-8.205749	-4.242749	-0.986366
68	6	-4.300923	-2.756516	-0.105965
69	6	-5.671238	-3.388551	-0.259201
70	6	-7.572326	-4.804360	0.121345
71	6	-6.289396	-4.374098	0.488598
72	1	-8.069858	-5.578958	0.697064
73	1	-5.789414	-4.814309	1.348164
74	6	-4.194840	-2.052052	1.260088
75	1	-3.206445	-1.617247	1.429603
76	1	-4.374425	-2.777734	2.060497
77	1	-4.941047	-1.255641	1.344325
78	8	1.074080	7.128943	-1.823685
79	6	0.297534	8.048150	-1.508744
80	8	0.037076	9.111816	-2.132686
81	6	-0.434969	7.891282	-0.137205
82	7	-1.418884	9.023189	-0.054486
83	1	0.302448	8.059869	0.652238
84	16	0.050914	1.980186	0.279793
85	6	-1.101776	6.535632	0.046770
86	1	-0.327000	5.784058	-0.129832
87	6	-1.710492	6.333669	1.443592
88	1	-1.869477	6.389616	-0.723344
89	6	-2.262866	4.925390	1.550170
90	1	-2.542339	7.023552	1.618941
91	1	-0.950344	6.520097	2.209621
92	8	-3.340257	4.609877	1.036669
93	7	-1.470452	4.035389	2.181987
94	6	-1.783001	2.627549	2.289832
95	1	-0.630007	4.333177	2.664858
96	7	-1.070670	0.764953	3.716685
97	1	-2.827157	2.516720	2.608690
98	6	-1.654306	1.910132	0.937478
99	1	-1.946302	0.862941	1.040989
100	1	-2.342373	2.373446	0.228133
101	6	-0.872252	2.047771	3.388638
102	8	-0.017989	2.756488	3.931247
103	1	-1.716583	0.177282	3.200756
104	6	-0.192145	0.056510	4.627333
105	1	0.836270	0.399548	4.480995
106	1	-0.458339	0.267942	5.669747
107	6	-0.232473	-1.463229	4.403852
108	8	-1.100823	-1.904984	3.584706
109	8	0.599752	-2.139183	5.035089
110	1	-0.479250	-2.842955	2.417299
111	1	-1.406855	9.524690	0.834027
112	1	-2.379877	8.728897	-0.239745
113	1	-1.932006	-1.252279	-0.052738
114	1	0.971179	-2.312328	-1.499154
115	6	-5.874783	-1.093357	-3.114528
116	6	-5.405249	-1.755071	-4.408797
117	1	-6.962617	-0.980887	-3.114335
118	1	-4.316143	-1.865346	-4.418936
119	1	-5.698624	-1.147603	-5.270858
120	1	-5.852818	-2.748437	-4.517657

121	1	-5.463575	-0.083650	-3.018967
122	1	6.926445	-3.374258	2.019556
123	1	-1.126660	9.653517	-0.833367

1-GSH *cyclic* E(RωB97XD) = -2796.2942764 Ha

Center Number	Atomic Number	Coordinates (Angstroms) X	Y	Z
1	1	0.392346	-2.187775	-2.603838
2	1	4.815273	-3.427146	-1.976874
3	1	6.413096	-3.961864	-1.417879
4	1	0.169798	-1.763258	-4.305902
5	6	5.888627	-3.386774	-2.188027
6	1	4.161603	-1.719863	1.811858
7	1	-1.872678	-1.320788	-2.962717
8	6	0.245544	-1.347647	-3.294998
9	6	-1.043812	-0.609973	-2.950903
10	1	3.173759	-0.248755	1.709614
11	6	4.187782	-0.626819	1.861361
12	1	4.496761	-0.335309	2.870739
13	1	3.954573	-1.084185	-2.346461
14	6	6.386154	-1.942784	-2.211773
15	6	1.533184	0.168027	-1.802157
16	1	-1.248712	0.130156	-3.740232
17	6	3.889385	-0.590852	-1.383114
18	1	8.785526	-2.351884	-0.713449
19	6	2.645446	0.062179	-1.042138
20	7	6.169732	-1.253046	-0.954344
21	6	0.263197	0.855974	-1.297747
22	6	4.985440	-0.636829	-0.579057
23	6	8.381189	-1.781500	0.116692
24	1	7.457229	-1.910228	-2.429648
25	6	7.106465	-1.224449	0.077825
26	1	2.608347	0.512547	-0.059606
27	1	10.135854	-2.016933	1.332000
28	6	9.137696	-1.591353	1.278598
29	6	5.179116	-0.050591	0.833019
30	6	6.585937	-0.514921	1.161483
31	16	0.464220	1.537163	0.404620
32	6	8.633710	-0.874069	2.362618
33	6	7.342404	-0.331404	2.304738
34	1	9.238703	-0.739848	3.254218
35	6	5.122560	1.489443	0.817636
36	1	4.125254	1.855027	0.555844
37	1	5.376194	1.876950	1.810310
38	1	5.838669	1.896227	0.096544
39	6	-0.091314	0.351323	1.578237
40	7	-0.962506	0.861240	2.481234
41	6	-1.359091	0.188250	3.575257
42	6	-0.903214	-1.095847	3.792804
43	6	-0.059446	-1.679349	2.846000
44	6	0.354407	-0.954051	1.733061
45	1	-2.047143	0.704401	4.232310
46	1	-1.222680	-1.636498	4.674592
47	1	0.292119	-2.695983	2.984537

48	1	6.067903	-3.863204	-3.157099
49	1	-3.580479	-2.706844	1.028967
50	1	2.355562	-0.908735	-3.473969
51	6	1.432486	-0.396078	-3.197757
52	1	-2.505712	-1.329710	1.294649
53	6	-3.502691	-1.724698	1.505930
54	1	-3.603830	-1.857455	2.588140
55	1	-3.581036	-0.359898	-2.385207
56	6	-1.014619	0.093360	-1.605680
57	1	1.297292	0.416929	-3.925440
58	6	-3.446215	-0.301918	-1.310486
59	1	-8.364057	-2.112285	-1.170148
60	6	-2.142172	0.168379	-0.859142
61	7	-5.748341	-0.992156	-1.069688
62	6	-4.513551	-0.641509	-0.543011
63	1	5.896401	-1.372379	-3.007016
64	6	-7.876996	-2.012300	-0.205261
65	6	-6.595510	-1.485789	-0.077112
66	1	-9.529469	-2.841310	0.887616
67	6	-8.528500	-2.425677	0.962709
68	6	-4.591661	-0.777917	0.982938
69	6	5.968677	-1.383979	1.166860
70	6	-7.917141	-2.318877	2.211061
71	6	-6.621962	-1.791572	2.315324
72	1	-8.441767	-2.648453	3.102790
73	1	-6.140064	-1.709222	3.286707
74	6	-4.531355	0.595460	1.675722
75	1	-3.592674	1.116839	1.478583
76	1	-4.638751	0.467988	2.758798
77	1	-5.346158	1.237691	1.325550
78	16	0.186881	2.529344	-2.229401
79	7	-0.194995	5.278394	-0.777230
80	6	-1.360387	4.358408	-0.610361
81	1	0.035622	5.603331	0.179773
82	1	-2.259683	4.968629	-0.737179
83	6	-1.396238	3.282083	-1.709757
84	1	-2.084223	2.490472	-1.400426
85	1	-1.804907	3.725598	-2.621307
86	6	-1.367175	3.924099	0.891051
87	8	-2.178442	3.023411	1.218935
88	8	-0.596829	4.567659	1.636117
89	1	-1.387379	1.791413	2.255705
90	1	-2.110036	0.690450	0.090877
91	1	1.033126	-1.376954	1.001208
92	6	-6.069580	-1.031089	-2.483180
93	6	-5.664633	-2.345579	-3.148571
94	1	-7.146170	-0.865907	-2.585071
95	1	-4.587294	-2.516566	-3.055883
96	1	-5.920166	-2.323735	-4.212727
97	1	-6.187025	-3.189231	-2.685589
98	1	-5.582111	-0.179815	-2.967867
99	1	6.944315	0.223859	3.150818
100	1	-0.374576	6.069345	-1.397250
101	1	0.622036	4.765752	-1.131844

1-GSH *acyclic* E(RωB97XD) = -2796.2958988 Ha

Center Number	Atomic Number	Coordinates (Angstroms) X	Y	Z
1	1	0.143219	-0.115317	3.388579
2	1	-4.534741	-1.458113	3.653244
3	1	-6.085706	-2.315046	3.547948
4	1	0.110877	1.353457	4.368990
5	6	-5.621809	-1.332065	3.679874
6	1	-3.596974	-3.116813	-0.080798
7	1	2.260309	1.142622	3.133189
8	6	0.121981	0.981194	3.338760
9	6	1.364926	1.495886	2.617905
10	1	-2.487963	-1.966519	-0.839604
11	6	-3.497912	-2.379845	-0.883735
12	1	-3.614246	-2.894918	-1.843135
13	1	-3.647775	0.408044	2.250802
14	6	-6.087145	-0.368225	2.590118
15	6	-1.153492	0.961764	1.173483
16	1	1.377445	2.593791	2.682758
17	6	-3.492859	0.059585	1.236085
18	1	-8.345383	-1.877968	1.699622
19	6	-2.223767	0.357776	0.611965
20	7	-5.739835	-0.818867	1.255695
21	6	0.106925	1.302214	0.386284
22	6	-4.516403	-0.610932	0.640342
23	6	-7.854704	-2.080963	0.753088
24	1	-7.172810	-0.243035	2.627483
25	6	-6.577623	-1.607981	0.467713
26	1	-2.142393	0.079544	-0.430647
27	1	-9.494885	-3.226723	-0.026603
28	6	-8.496984	-2.846679	-0.226454
29	6	-4.561388	-1.274819	-0.748999
30	6	-5.945214	-1.896358	-0.742676
31	16	0.145768	0.583057	-1.326734
32	6	-7.880328	-3.131658	-1.443981
33	6	-6.588787	-2.653173	-1.705001
34	1	-8.397816	-3.728819	-2.188641
35	6	-4.449458	-0.249795	-1.894024
36	1	-3.453928	0.199367	-1.951417
37	1	-4.644893	-0.746989	-2.850095
38	1	-5.182573	0.553119	-1.768510
39	6	0.116293	-1.162517	-1.120746
40	7	-0.036916	-1.870089	-2.266323
41	6	-0.068704	-3.219122	-2.320454
42	6	0.051849	-3.950855	-1.163734
43	6	0.210641	-3.262755	0.042503
44	6	0.246084	-1.876295	0.069260
45	1	-0.195790	-3.651280	-3.303867
46	1	0.022184	-5.031868	-1.204039
47	1	0.310329	-3.812743	0.971916
48	1	-5.900571	-0.948118	4.666286
49	1	4.055608	-2.689832	-0.264921
50	1	-2.022680	1.082810	3.134050
51	6	-1.136601	1.439068	2.606036

52	1	2.923980	-1.580405	-1.055321
53	6	3.954662	-1.942575	-1.058220
54	1	4.135352	-2.433393	-2.020343
55	1	3.959400	0.720063	2.198460
56	6	1.407813	1.077258	1.165075
57	1	-1.177088	2.538849	2.628028
58	6	3.821077	0.393833	1.173795
59	1	8.786386	-1.253511	1.554554
60	6	2.525993	0.608112	0.569242
61	7	6.120962	-0.335781	1.152256
62	6	4.882080	-0.187387	0.550492
63	1	-5.660245	0.628403	2.738691
64	6	8.302440	-1.458226	0.604916
65	6	6.998325	-1.052613	0.338666
66	1	10.001536	-2.484622	-0.214036
67	6	8.982159	-2.157555	-0.398770
68	6	4.961781	-0.794541	-0.863082
69	6	6.376346	-1.342824	-0.876748
70	6	8.375894	-2.443114	-1.621335
71	6	7.057426	-2.032743	-1.863225
72	1	8.922662	-2.987876	-2.384997
73	1	6.579961	-2.258197	-2.813898
74	6	4.804902	0.275776	-1.960814
75	1	3.796914	0.699612	-1.980193
76	1	4.999196	-0.173700	-2.940558
77	1	5.518219	1.092177	-1.810016
78	16	0.133278	3.142356	-0.030227
79	7	-0.977388	4.287178	-2.771447
80	6	-1.678605	4.631883	-1.496178
81	1	-0.591964	5.191354	-3.107565
82	1	-2.729819	4.795890	-1.746622
83	6	-1.602805	3.498601	-0.482220
84	1	-2.164401	3.794546	0.404768
85	1	-2.058825	2.593686	-0.892434
86	6	-1.100473	6.002680	-1.014529
87	8	-1.416926	6.351079	0.136923
88	8	-0.409671	6.612217	-1.871101
89	1	-0.141090	-1.365519	-3.143669
90	1	2.454250	0.366548	-0.483330
91	1	0.376631	-1.344799	1.001233
92	6	6.445223	0.083420	2.502616
93	6	6.054741	-0.953945	3.553640
94	1	7.519825	0.282393	2.541019
95	1	4.978775	-1.153542	3.525546
96	1	6.312620	-0.592145	4.554012
97	1	6.584509	-1.896337	3.379977
98	1	5.951001	1.040863	2.693254
99	1	-6.102447	-2.878958	-2.651061
100	1	-1.579384	3.860504	-3.476322
101	1	-0.181498	3.662513	-2.599824

Cy7-PyrH E(RPBE0) = -2073.7125673 Ha

Center Number	Atomic Number	Coordinates (Angstroms) X	Y	Z

1	1	0.025392	3.752891	-0.890946
2	1	5.498401	3.649590	-0.626876
3	1	7.246647	3.378413	-0.707882
4	1	-6.643093	4.486491	0.176176
5	1	-0.021422	4.600776	0.649515
6	6	6.406625	3.543341	-0.030422
7	1	5.029661	0.000833	-2.709181
8	1	-2.145877	3.381436	0.131579
9	6	-0.014799	3.608243	0.193848
10	6	-1.283514	2.861607	0.555688
11	1	3.520247	-0.752317	-2.172577
12	6	4.607634	-0.816704	-2.121510
13	1	4.911578	-1.760075	-2.580066
14	1	3.749726	2.238937	0.645573
15	6	6.278112	2.404848	0.966185
16	6	1.217717	1.431545	0.095953
17	1	-1.424165	2.867282	1.644295
18	6	3.683758	1.256394	0.196471
19	1	8.891918	1.393304	0.562012
20	6	2.425486	0.759901	-0.109926
21	7	6.071321	1.117606	0.320000
22	6	-0.025058	0.825188	-0.163834
23	6	4.885136	0.589521	-0.026886
24	1	-5.520435	2.686416	1.488645
25	6	8.494584	0.494363	0.106900
26	1	7.179056	2.315423	1.574274
27	6	7.135474	0.285195	-0.054284
28	1	2.358643	-0.231216	-0.538047
29	1	10.417852	-0.375265	-0.241634
30	6	9.347082	-0.504948	-0.350048
31	6	5.116717	-0.773612	-0.674227
32	6	6.623010	-0.859274	-0.648947
33	16	-0.026226	-0.812602	-0.896310
34	6	8.850464	-1.660031	-0.942481
35	6	7.477876	-1.845013	-1.096549
36	1	9.537649	-2.422752	-1.290055
37	1	7.096556	-2.748167	-1.561407
38	6	4.514385	-1.906364	0.170441
39	1	3.425219	-1.863552	0.187711
40	1	4.808469	-2.867347	-0.256664
41	1	4.877195	-1.862966	1.199104
42	6	0.071052	-1.853654	0.500327
43	7	0.150706	-3.174532	0.240461
44	6	0.228021	-4.119113	1.194875
45	6	0.230969	-3.756528	2.513454
46	6	0.149763	-2.400203	2.825838
47	6	0.069186	-1.449361	1.828662
48	1	0.287348	-5.140541	0.846208
49	1	0.294704	-4.515396	3.280187
50	1	0.148039	-2.082966	3.861873
51	1	0.003409	-0.396051	2.062243
52	1	-5.556335	3.571310	-0.879684
53	1	-7.303467	3.288320	-0.945753
54	1	6.577318	4.478792	0.505231
55	6	-6.467002	3.511059	-0.280499

56	1	-5.076875	-0.200620	-2.665367
57	1	2.111943	3.359802	0.313367
58	6	1.211610	2.835983	0.640660
59	1	-3.558469	-0.894146	-2.076795
60	6	-4.644902	-0.969255	-2.021692
61	1	-4.936755	-1.946463	-2.412145
62	1	-3.805953	2.273393	0.530406
63	6	-6.339247	2.458226	0.806260
64	6	-1.269628	1.436343	0.066697
65	1	1.252939	2.800923	1.737078
66	6	-3.734734	1.262128	0.151149
67	1	-8.946162	1.403052	0.476353
68	6	-2.474821	0.749589	-0.113943
69	7	-6.122933	1.124236	0.266296
70	6	-4.933522	0.576587	-0.032339
71	1	5.454493	2.573498	1.660231
72	6	-8.542628	0.474421	0.091860
73	1	-7.243359	2.413506	1.414406
74	6	-7.181975	0.260479	-0.048013
75	1	-2.401191	-0.265668	-0.479696
76	1	-10.460063	-0.429024	-0.197613
77	6	-9.388235	-0.560797	-0.291958
78	6	-5.155750	-0.830417	-0.580783
79	6	-6.661361	-0.922712	-0.552736
80	6	-8.883451	-1.754470	-0.794320
81	6	-7.509441	-1.943577	-0.928863
82	1	-9.565548	-2.544586	-1.086259
83	1	-7.121483	-2.876807	-1.323515
84	6	-4.547259	-1.899042	0.339332
85	1	-3.458124	-1.852460	0.350236
86	1	-4.839771	-2.888082	-0.019342
87	1	-4.907996	-1.785610	1.363409
88	1	0.154111	-3.481172	-0.726996

Cy7-SMe E(RPBE0) = -1865.6770065 Ha

Center Number	Atomic Number	Coordinates (Angstroms) X	Y	Z
1	1	-0.323483	3.116373	1.571349
2	1	-5.616632	3.175849	0.677237
3	1	-7.360721	2.876910	0.609147
4	1	6.642765	3.860416	1.360342
5	1	-0.066747	4.320283	0.314000
6	6	-6.483343	3.138458	0.014310
7	1	-5.198634	-0.696882	2.295719
8	1	1.995774	2.966456	0.918990
9	6	-0.094914	3.245428	0.507898
10	6	1.251464	2.624873	0.192258
11	1	-3.635973	-1.343087	1.771398
12	6	-4.715533	-1.423414	1.639396
13	1	-5.021764	-2.423872	1.952909
14	1	-3.754935	1.975618	-0.651475
15	6	-6.270191	2.140421	-1.110547
16	6	-1.239715	1.106676	-0.027965

17	1	1.600666	2.977576	-0.786983
18	6	-3.698951	0.944442	-0.325669
19	1	-8.878124	1.031106	-1.017627
20	6	-2.452132	0.420249	-0.002398
21	7	-6.072143	0.784053	-0.626343
22	6	-0.018777	0.440798	0.202349
23	6	-4.892326	0.237824	-0.271084
24	1	5.472653	2.834225	-0.588156
25	6	-8.484279	0.088049	-0.658785
26	1	-7.131023	2.119674	-1.780173
27	6	-7.132248	-0.111346	-0.436053
28	1	-2.387449	-0.619916	0.288185
29	1	-10.400358	-0.858791	-0.557109
30	6	-9.335375	-0.980330	-0.394591
31	6	-5.128041	-1.203633	0.177526
32	6	-6.626857	-1.314491	0.037699
33	16	-0.039856	-1.330795	0.448227
34	6	-8.845179	-2.192990	0.074386
35	6	-7.479860	-2.367391	0.294697
36	1	-9.530541	-3.008943	0.272764
37	1	-7.101865	-3.315544	0.662894
38	6	-4.437848	-2.207844	-0.757655
39	1	-3.351187	-2.147607	-0.686991
40	1	-4.739341	-3.220656	-0.481460
41	1	-4.727048	-2.036865	-1.796488
42	6	0.495323	-1.457224	2.177101
43	1	5.562232	2.577469	1.925614
44	1	7.305870	2.285789	1.820560
45	1	-6.638434	4.134397	-0.404535
46	6	6.460984	2.784302	1.341280
47	1	5.069643	-1.592415	1.866024
48	1	-2.150263	3.044298	-0.088596
49	6	-1.187154	2.583642	-0.314563
50	1	3.528525	-1.968131	1.078314
51	6	4.612138	-1.999409	0.962208
52	1	4.909701	-3.046198	0.869667
53	1	3.763723	2.052636	0.143142
54	6	6.298819	2.320101	-0.095706
55	6	1.220615	1.116362	0.184445
56	1	-1.000271	2.752937	-1.383098
57	6	3.685446	0.977122	0.042407
58	1	8.890506	1.200806	-0.369242
59	6	2.415518	0.408994	0.091647
60	7	6.068845	0.888613	-0.199231
61	6	4.869230	0.275687	-0.133822
62	1	-5.410278	2.411401	-1.724026
63	6	8.473147	0.203188	-0.430588
64	1	7.192203	2.545130	-0.679633
65	6	7.111297	-0.034259	-0.349026
66	1	2.319071	-0.666529	0.009700
67	1	10.373902	-0.752773	-0.656879
68	6	9.302237	-0.902611	-0.590032
69	6	5.074239	-1.231038	-0.283448
70	6	6.575805	-1.313544	-0.412989
71	6	8.781761	-2.188945	-0.661992
72	6	7.407162	-2.402378	-0.572529

73	1	9.450230	-3.033075	-0.786273
74	1	7.005155	-3.408780	-0.627472
75	6	4.403401	-1.770716	-1.554806
76	1	3.316052	-1.711078	-1.493750
77	1	4.678984	-2.819081	-1.689225
78	1	4.731073	-1.215202	-2.435729
79	1	0.506129	-2.522904	2.407927
80	1	-0.217578	-0.952348	2.827592
81	1	1.493383	-1.044935	2.316692

Cy7-SMe E(RPBE0) = -1522.922675 Ha

Center Number	Atomic Number	Coordinates (Angstroms) X	Y	Z
1	1	0.750292	2.794225	1.475483
2	1	-5.420815	2.132839	2.483537
3	1	-7.180686	1.982429	2.359895
4	1	6.312832	4.063760	-1.254670
5	1	0.152854	3.989810	0.331021
6	6	-6.306962	2.505597	1.965876
7	1	-5.235243	-1.975375	1.614614
8	1	2.005650	2.716692	-0.683257
9	6	0.227628	2.916043	0.521180
10	6	1.039242	2.226336	-0.568696
11	1	-3.698232	-2.255583	0.786716
12	6	-4.779558	-2.221416	0.653423
13	1	-5.117019	-3.217981	0.359817
14	1	-3.678732	1.838774	0.621561
15	6	-6.187203	2.308843	0.464316
16	6	-1.200221	0.818901	0.374770
17	1	0.517918	2.316886	-1.529951
18	6	-3.665259	0.805916	0.296314
19	1	-8.846798	1.418540	0.038904
20	6	-2.412604	0.181480	0.200781
21	7	-6.050600	0.913672	0.088007
22	6	0.017764	0.092114	0.207829
23	6	-4.884685	0.227393	0.009946
24	1	5.276397	2.001214	-2.195879
25	6	-8.492599	0.429692	-0.226178
26	1	-7.068967	2.696620	-0.048193
27	6	-7.146793	0.102160	-0.218456
28	1	-2.384020	-0.861407	-0.086682
29	1	-10.451123	-0.346870	-0.601254
30	6	-9.390428	-0.571381	-0.585215
31	6	-5.189036	-1.207443	-0.423004
32	6	-6.693993	-1.170224	-0.542599
33	7	0.056492	-1.249869	0.349417
34	6	-8.952551	-1.846859	-0.918288
35	6	-7.592595	-2.154381	-0.896915
36	1	-9.672846	-2.608567	-1.193785
37	1	-7.253783	-3.152738	-1.154559
38	6	-4.557402	-1.541281	-1.780997
39	1	-3.468382	-1.568247	-1.727159
40	1	-4.904154	-2.523638	-2.109751

41	1	-4.845846	-0.807081	-2.535857
42	1	5.367046	3.251741	0.001951
43	1	7.131751	3.111056	-0.008473
44	1	-6.414197	3.568392	2.191185
45	6	6.244051	3.162484	-0.642361
46	1	5.258319	-0.243461	2.380094
47	1	-1.632606	2.557358	1.556369
48	6	-1.166342	2.312293	0.594888
49	1	3.764238	-1.109544	1.996224
50	6	4.848897	-1.124285	1.881490
51	1	5.230386	-2.012888	2.389756
52	1	3.655241	1.664579	-1.027673
53	6	6.144239	1.938645	-1.537227
54	6	1.213635	0.786254	-0.179785
55	1	-1.795868	2.782803	-0.170352
56	6	3.676203	0.714729	-0.508225
57	1	8.836172	1.099864	-1.220285
58	6	2.444871	0.179319	-0.086871
59	7	6.056793	0.698398	-0.790027
60	6	4.910838	0.136547	-0.322530
61	1	-5.333012	2.855076	0.061003
62	6	8.516299	0.219061	-0.676760
63	1	7.017148	1.864359	-2.187950
64	6	7.180387	-0.043232	-0.419878
65	1	2.476681	-0.797729	0.382373
66	1	10.504164	-0.518063	-0.384889
67	6	9.450586	-0.696356	-0.200251
68	6	5.266480	-1.161807	0.406241
69	6	6.773008	-1.165064	0.291516
70	6	9.058031	-1.825632	0.506422
71	6	7.707132	-2.065969	0.757042
72	1	9.805627	-2.522499	0.867548
73	1	7.403044	-2.948410	1.310960
74	6	4.690206	-2.391762	-0.309038
75	1	3.601465	-2.421726	-0.256907
76	1	5.073771	-3.298372	0.164586
77	1	4.983972	-2.404951	-1.360418
78	6	-0.690676	-2.061931	1.287910
79	1	0.007244	-2.684746	1.851562
80	1	-1.402205	-2.723263	0.785813
81	1	-1.231700	-1.423872	1.984684
82	1	0.861674	-1.704127	-0.049516
